# Probability and heritability estimates on primary osteoarthritis of the hip leading to total hip arthroplasty: a nationwide population based follow-up study in Danish twins

**DOI:** 10.1186/s13075-015-0854-4

**Published:** 2015-11-20

**Authors:** Søren Glud Skousgaard, Jacob Hjelmborg, Axel Skytthe, Lars Peter Andreas Brandt, Sören Möller, Søren Overgaard

**Affiliations:** Department of Occupational and Environmental Medicine, Odense University Hospital, 5000 Odense C, Denmark; Department of Epidemiology, Biostatistics, and Biodemography, The Danish Twin Registry, Department of Public Health, University of Southern Denmark, 5000 Odense C, Denmark; Department of Orthopaedic Surgery and Traumatology & Orthopaedic Research Unit, Odense University Hospital, 5000 Odense C, Denmark; Institute of Clinical Research, University of Southern Denmark, 5000 Odense C, Denmark

**Keywords:** Hip osteoarthritis, Cumulative incidence, Twin study, Heritability, Total hip arthroplasty

## Abstract

**Introduction:**

Primary hip osteoarthritis, radiographic as well as symptomatic, is highly associated with increasing age in both genders. However, little is known about the mechanisms behind this, in particular if this increase is caused by genetic factors. This study examined the risk and heritability of primary osteoarthritis of the hip leading to a total hip arthroplasty, and if this heritability increased with increasing age.

**Methods:**

In a nationwide population-based follow-up study 118,788 twins from the Danish Twin Register and 90,007 individuals from the Danish Hip Arthroplasty Register for the period 1995 to 2010 were examined. Our main outcomes were the cumulative incidence, proband-wise concordance and heritability on age, within-pair correlations in monozygotic and dizygotic twin pairs, and the genetic and environmental influence estimated in models taking into account that individuals may not have had a total hip arthroplasty at the time of follow-up.

**Results:**

There were 94,063 twins eligible for analyses, comprising 835 cases of 36 concordant and 763 discordant twin pairs. The probability increased particularly from 50 years of age. After sex and age adjustment a significant additive genetic component of 47 % (12:79), a shared environmental component of 21 % (2:76) and a unique environment component of 32 % (21:41) accounted for the variation in population liability to total hip arthroplasty. The sex-adjusted proband-wise concordance and heritability on age indicated an increasing age-associated genetic influence onwards from 60 years of age.

**Conclusion:**

The cumulative incidence in primary hip osteoarthritis leading to total hip arthroplasty increases in particular after the age of 50 years in both genders. Family factors of genes and shared environment are highly significant and account for 68 % of the variation in the population liability to total hip arthroplasty; however, the genetic influence increases significantly from 60 years of age onwards.

## Introduction

Osteoarthritis (OA) is one of the major causes of mobility disability and loss of work days or employment causing substantial health and economic burdens to the individual as well as public healthcare resources [1, 2] Patients with primary OA of the hip are regularly encountered in the clinic; however, the radiographic findings may often be inconsistent with the clinical presentation [3–5]. From a clinical point of view, cases with nonsymptomatic radiographic hip OA are less relevant in contrast to cases with total hip arthroplasty (THA), as the latter represents a severe disease burden and a well-defined outcome [5, 6].

Although studies on hip OA prevalence, both radiographic and symptomatic, are numerous [7], studies on hip OA incidence are relatively few. Some examine hip OA incidence related to acetabular dysplasia or proximal femur shape, but only a few examine symptomatic hip OA incidence [8, 9].

Painful hip OA is a multifactorial complex disease considered to be caused by environmental and genetic factors [10]. Environmental risk factors frequently referred to include occupation, hip joint straining work tasks, body mass index (BMI), previous joint injury, and sports [11–15].

Sibling and family studies have pointed at a strong genetic and family predisposition, but these studies cannot discriminate genetic from common environmental components [16–18]. However, the classical twin design (CTD) offers an opportunity to differentiate the relative influence and magnitude of genetic and environmental components on a particular disease or trait [19]. Few twin studies on the heritability of hip OA have been published so far. In two studies on healthy female twins, MacGregor et al reported a genetic component in radiographic hip OA with a narrow sense heritability (variation caused by additive genetic components) of 58 and 28 %, respectively [20, 21]. In a twin study on white American ex-service men Page et al reported an additive genetic component accounting for 53 % in the liability to THA [22]. These twin studies were cross-sectional, the selected twin populations did not include both sexes and none reported the presence of a common environmental component in hip OA.

Patients with symptomatic and radiographic confirmed hip and knee OA are liable to considerable co-morbidity resulting in a higher mortality risk compared to that of the background population [23]. As this increased risk, as well as the age-related increase in symptomatic hip OA [24], may affect the heritability estimates, we decided to examine Danish twins in a nationwide population-based time-to-event analysis taking into account the competing risk of death in the population.

Accordingly, the aims of this study were to examine the probability and heritability of THA due to primary hip OA, both sex stratified as well as sex adjusted, in a competing risk setting, by means of cumulative incidence function, biometric modeling, and age-related cumulative heritability.

## Material and methods

The study participants were selected from two nationwide Danish registers: the Danish Twin Register (DTR) and the Danish Hip Arthroplasty Register (DHR).

The DTR was established in 1954 and comprises approximately 170,000 twins born since 1870 [25]. After adjustment for infant mortality the completeness of the twin ascertainment is high with approximately 90 % twins ascertained before and up to 1968, and complete ascertainment of all live born twin pairs since 1968 [26]. Zygosity of same-sex twins is assessed by a four-item questionnaire on the similarity of the two twins, which will classify their zygosity correctly in 95 % of all same-sex twin pairs [27]. All twin pairs in the DTR both alive at 1 January 1995 comprised the study base cohort. Information on sex, date of birth, zygosity, vital status, and date of death or censoring due to emigration or end of follow-up was obtained. Excluded from final analysis were twins with unknown or uncertain zygosity.

The DHR was established in 1995 and holds information on primary and revision arthroplasty performed in Denmark onwards from 1995. The recorded diagnosis for every surgical procedure is based on International Classification of Diseases 10 (ICD10). The diagnosis of hip OA has been validated with a positive predictive value (PPV) of 94 %, and a completeness of 92 to 96 % based on annual reports [28, 29]. The information used included diagnosis and date of operation from the period 1 January 1995 to 31 August 2010. Individuals with THA due to other causes were excluded. In individuals with more than one admission care was taken that a case was included only once; i.e., an individual with bilateral THA with two separate and subsequent recordings was not included twice. After exclusion the final twin study cohort was created by means of the Danish civil registration number by which the two data files were merged. The Danish civil registration number is unique to each Danish citizen comprising a ten digit number holding information on date of birth and sex of the individual. The final study cohort comprised monozygotic (MZ), same-sex dizygotic (SSDZ), and opposite-sex dizygotic (OSDZ) twins. A case was defined as a twin, who had had a THA with the recorded ICD10 diagnosis of M160 and M161, independently of co-twin status.

### Ethics

This study was reviewed and approved by The Regional Scientific Ethical Committees for Southern Denmark and The Danish Data Protection Agency, and permission was granted to use the relevant data from the DHR and the DTR with no patient consent needed.

### Statistical analyses

The analyses included descriptive summaries and biometric modeling of genetic and environmental components taking time to THA into account. The CTD is based on the assumption that MZ twins have identical genotypes, whereas both SSDZ and OSDZ twins share on average one-half of their segregating genes as ordinary siblings. If a markedly greater phenotypic similarity in MZ twin pairs compared to that of DZ twin pairs is observed, a genetic influence on the disease in question can be inferred [19]. The similarity in MZ and DZ twin pairs was assessed by means of case-wise and proband-wise concordance rates, and tetrachoric correlation coefficients, reflecting the magnitude of the relative influence of genetic and environmental effects [19, 30]. The concordance rates reflect the probability of one twin having the disease in question, conditional that the co-twin is affected. A phenotypic variation in a twin population can be separated into additive (A) and nonadditive (D) genetic variation, and common (C) and individual (E) environmental variation based on the defined underlying correlation structure in a twin population [19]. Based on the polygenetic liability-threshold model [30], broad sense heritability (*H*^*2*^) is a measure of the proportion of the variance in liability to a disease or trait caused by additive and nonadditive genetic effects.

The sex-stratified and sex-adjusted model fitting included saturated models, and submodels composed of the variance components ACE, ADE, AE and CE. As for comparison between the non-nested models, the Akaike’s Information Criterion was used, and for comparison between nested models the log likelihood ratio test was used.

The cumulative incidence function (CIF) is estimated for the competing risk situation [31, 32]. If an individual is at risk of experiencing more than one event, each affecting the other, these events are termed competing risks. We used CIF to estimate the probability of getting a THA, adjusted for the competing risk of death, by means of an illness–death model with three states corresponding to “healthy”, “diseased” and “dead”. The transition probabilities describe the probability that a healthy individual at a later time will be diseased or dead [33]. In a time-to-event analysis with competing risks only a proportion of the observations are known at follow-up, individuals appearing as noncases may become cases, but at a future time point we do not know, or may be lost to follow-up due to emigration. This is termed right censoring, and we used the liability-threshold modeling with inverse probability weighting analyzing the twin data [34–36]. The analyses were carried out using bivariate probit models for twin data implemented in the R Mets-package (https://cran.r-project.org/web/packages/mets/index.html). For comparisons between groups a two-tailed *t* test or likelihood ratio chi-squared test was used as appropriate. A *p*-value equal or less than 0.05 was considered significant and confidence intervals (CI) were expressed as 95 % CI. Calculations were carried out in the statistical software R and Stata11.

## Results

**Fig. 1:**
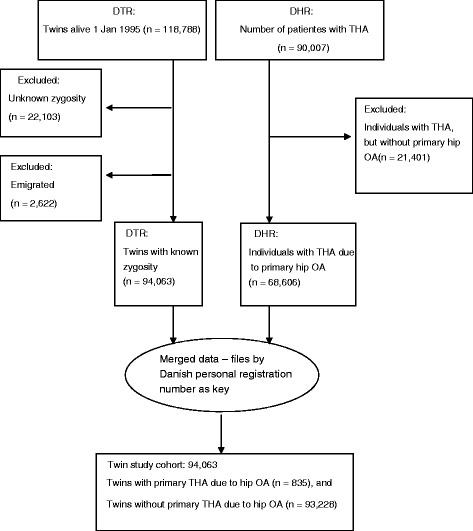
Flow chart of the participating twins. *DHR* Dansih Hip Arthroplasty Register, *DTR* Danish Twin Register, *OA* Osteoarthritis, *THA* Total hip arthroplasty

The 1995 twin cohort comprised 59,394 twin pairs alive in 1995. The DHR, by 31 August 2010, comprised the records of 90,007 individuals of which 68,606 individuals had primary hip OA (76 %). The number of twins that had a record in the DHR was 1196; of these twins, 917 (77 %) had the diagnosis of primary hip OA. After exclusion, the final study cohort comprised 94,063 twins of whom 835 were cases comprising 36 concordant (both twins are affected) and 763 discordant twin pairs (only one is affected) (Fig. 1). In 41,856 twin pairs both twins were alive at follow-up, while in 1891 twin pairs both twins had died, and in 6569 twin pairs only one twin was alive at follow-up. The fraction of the population censored comprised 84–85 % in MZ and SSDZ twins, and 91 % in OSDZ twins. Basic description and distribution by zygosity, THA, sex, and vital status is displayed in Table 1.

**Table 1:** Distribution by zygosity, sex, THA and vital status

THA by zygosity	Distribution by sex			Distribution by vital status				
Zygosity	Males (n)	Females (n) (%)	Total by sex (n)	Males alive (n)	Males dead (n)	Females alive (n)	Females dead (n)	Total by vital status (n)
MZ + THA	88	90 (51)	178	71	17	72	18	178
MZ – THA	9861	10,288 (51)	20,149	8671	1190	9059	1229	20,149
DZ + THA	184	189 (51)	373	141	43	155	34	373
DZ – THA	18,015	16,975 (49)	34,990	15,417	2598	14,486	2489	34,990
OS + THA	135	149 (52)	284	120	16	136	12	284
OS – THA	19,005	19,084 (50)	38,089	17,491	1514	17,892	1192	38,089
Total	47,288	46,775 (50)	94,063	41,911	5378	41,799	4974	94,063

*DZ* same-sex dizygotic, *MZ* monozygotic, *OS* opposite-sex dizygotic, *+ THA* Total hip arthroplasty, *– THA* No hip arthroplasty

The twin cases comprised 428 females and 407 males with THA, *p* = 0.2. Mean age was significantly higher in females (66.7 years; 66.8:68.6) compared to males (65.7 years; 64.8:66.6; range 33 to 94 years of age in both genders; *p* = 0.002).

### Concordance and tetrachoric correlations

When including only complete pairs alive at follow-up, the differences in MZ and DZ pairs with respect to the number of concordant pairs indicated a genetic component in both genders. These findings were supported by the difference in tetrachoric correlation coefficients in MZ and DZ twin pairs of both genders further indicating the presence of a genetic component in both females and males. The correlation in the SSDZ twin pairs did not exceed that of the OSDZ twin pairs (Table 2).

**Table 2:** Concordance and tetrachoric correlations

Zygosity	Concordant pairs with THA due to OA n (%)	Discordant pairs with THA due to OA n (%)	Concordant pairs without THA n (%)	Total twin pairs in zygosity groups n (%)	Tertrachoric correlations *(rho)*
MZM	10 (0.2)	57 (1.2)	4558 (98.6)	4625 (100)	0.70
DZM	5 (0.1)	148 (1.8)	8107 (98.1)	8260 (100)	0.33
MZF	9 (0.2)	72 (1.5)	4773 (98.3)	4855 (100)	0.66
DZF	5 (0.1)	148 (1.9)	7740 (98.0)	7893 (100)	0.33
OSDZ	7 (0.05)	223 (1.25)	17,602 (98.7)	17,832 (100)	0.41

*DZF* dizygotic females, *DZM* dizygotic males, *MZF* monozygotic females, *MZM* monozygotic males, *OA* Osteoarthritis, *OSDZ* opposite-sex dizygotic twin pairs, *THA* Total hip arthroplasty

### Cumulative incidence

**Fig. 2:**
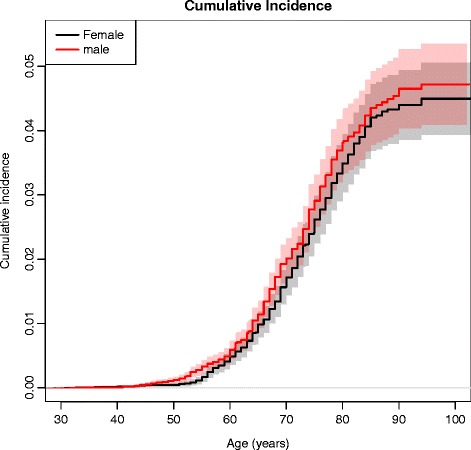
Cumulative incidence. Cumulative incidence for THA in males (*red line*) and females (*black line*)

From the age of 50 years an incidence increase was detectable in both genders peaking four- to fivefold at the age of 85 years without significant sex difference (Fig. 2).

### Proband-wise concordance rates on age

**Fig. 3:**
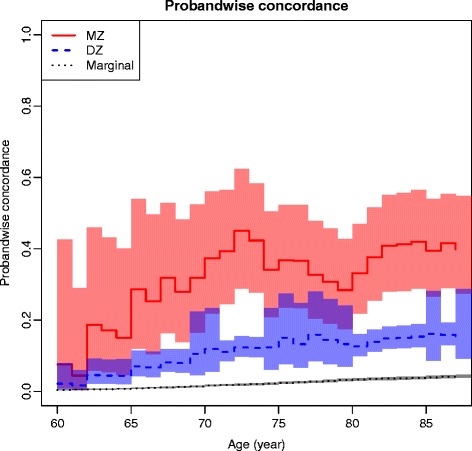
Proband-wise concordance rate by age in THA, sex-adjusted. *DZ* Dizygotic, *Marginal* Background incidence, *MZ* Monozygotic

Figure 3 displays the sex-adjusted proband-wise condordance rate, expressed as age-dependent increase in the MZ–DZ differences, indicating an increasing genetic influence.

### Prevalences, tetrachoric correlations and case-wise concordance rates

Equal prevalence in MZ and DZ twin pairs in all models was observed. In all models estimating a genetic component, sex-stratified and sex-adjusted, both tetrachoric correlations and case-wise concordant rates were markedly higher in MZ compared to DZ twins, underlining the presence of a genetic component (Table 3).

**Table 3:** Prevalences, tetrachorics, case-wise concordance rates, heritability, biometric models and chi-squared test statistics

Model	MZ prevalence	DZ prevalence	MZr	DZr	MZcw	DZcw	Heritability	Variance component estimates				Chisquare test statistics				AIC
								A	D	C	E	LL	Chisquare	df	*p*	
Men																
Saturated	0.06	0.06	0.80 (0.57–0.91)	0.41 (0.09–0.65)	0.51 (0.33–0.70)	0.21 (0.10–0.40)	0.78 (0.07–0.99)	–	–	–	–	–621.44		6		1254.88
ACE	0.06	0.06	0.80 (0.57–0.91)	0.41 (0.09–0.65)	0.51 (0.33–0.70)	0.21 (0.10–0.40)	0.78 (0.07–0.99)	0.78 (0.07–0.99)	–	0.02 (0.00–1.0)	0.20 (0.08–0.41)	–621.44		6		1254.88
ADE	0.06	0.06	0.80 (0.58–0.91)	0.40 (0.32–0.47)	0.51 (0.34–0.69)	0.21 (0.16–0.26)	0.80 (0.60–0.91)	0.80 (0.60–0.91)	0.00 (0.00–1.00)	–	0.20 (0.09–0.40)					1255.30
AE	0.06	0.06	0.80 (0.58–0.91)	0.40 (0.32–0.47)	0.51 (0.34–0.69)	0.21 (0.16–0.26)	0.80 (0.60–0.91)	0.80 (0.60–0.91)	–	–	0.20 (0.09–0.40)	–621.65	0.42	2	0.81	
CE	0.07	0.07	0.63 (0.44–0.76)	0.63 (0.44–0.76)	0.37 (0.26–0.50)	0.37 (0.26–0.50)	–	–	–	0.63 (0.46–0.77)	0.37 (0.23–0.54)	–2921.4	4600	2	<0.0001	
Women																
Saturated	0.06	0.06	0.72 (047–0.87)	0.46 (0.15–0.69)	0.42 (0.25–0.61)	0.23 (0.11–0.43)	0.52 (0.07–0.94)	–	–	–	–	–621.44		6		1254.88
ACE	0.06	0.06	0.72 (0.47–0.87)	0.46 (0.15–0.69)	0.42 (0.25–0.61)	0.23 (0.11–0.43)	0.52 (0.07–0.94)	0.52 (0.07–0.94)	–	0.20 (0.006–0.91	0.28 (0.13–0.50)	–621.44		6		1254.88
ADE	0.06	0.06	0.74 (0.52–0.87)	0.37 (0.28–0.45)	0.44 (0.28–0.61)	0.18 (0.14–0.24)	0.74 (0.54–0.87)	0.74 (0.54–0.87)	0.00 (0.00–1.00)		0.26 (0.13–0.46)					1255.30
AE	0.06	0.06	0.74 (0.52–0.87)	0.37 (0.28–0.45)	0.44 (0.28–0.61)	0.18 (0.14–0.24)	0.74 (0.54–0.87)	0.74 (0.54–0.87)	–	–	0.26 (0.13–0.46)	–621.65	0.42	6	0.81	
CE	0.07	0.07	0.66 (0.48–0.79)	0.66 (0.48–0.79)	0.37 (0.26–0.50)	0.37 (0.26–0.50)	–	–	–	0.66 (0.50–0.79)	0.34 (0.21–0.50)	–2921.4	4600	2	<0.0001	
Sex-adjusted																
Saturated	0.06	0.06	0.68 (0.55–078)	0.45 (0.23–0.62)	0.39 (0.30–0.50)	0.23 (0.14–0.36)	0.47 (0.12–0.85)	–	–	–	–	–806.7		2		1621.34
ACE	0.06	0.06	0.68 (0.55–0.78)	0.45 (0.23–0.62)	0.39 (0.30–0.50)	0.23 (0.14–0.36)	0.47 (0.12–0.85)	0.47 (0.12–0.85)	–	0.22 (0.02–0.76)	0.31 (0.21–0.44)	–806.7	–	4		1621.34
ADE	0.06	0.06	0.70 (0.57–0.80)	0.35 (0.29–0.40)	0.41 (0.31–0.51)	0.18 (0.15–0.21)	0.70 (0.58–0.79)	0.70 (0.58–0.80)	0.00 (0.00–0.00)	–	0.30 (0.21–0.42)					1622.31
AE	0.06	0.06	0.70 (0.58–0.79)	0.35 (0.29–0.40)	0.41 (0.31–0.51)	0.18 (0.15–0.21)	0.70 (0.58–0.79)	0.70 (0.58–0.79)	–	–	0.30 (0.21–0.42)	–807.2	0.97	1	0.325	
CE	0.07	0.07	0.62 (0.51–0.71)	0.62 (0.51–0.71)	0.37 (0.29–0.45)	0.37 (0.29–0.45)	–	–	–	0.62 (0.52–0.72)	0.38 (0.28–0.48)	–2913.8	4214	1	<0.0001	

*A* Additive genetic, *AIC* Akaiki’s information criterion, *C* Common environment, *D* Dominant genetic, *DZcw* Dizygotic case-wise concordance rate, *DZr* Dizygotic correlation, *E* Unique environment, *MZcw* Monozygotic case-wise concordance rate, *MZr* Monozygotic correlation, *Saturated* Saturated model not including censoring

#### Biometric modeling

The first model in Table 3 displays the broad sense heritability in the saturated model (all co-variances are treated as free parameters), reflecting the proportion of variance attributable to genetic factors. Sex-stratified, the genetic factors seem to be more pronounced in males than in females; however, sex adjustment reduced the broad sense heritability estimate to 47 %. In the sex-adjusted saturated model the DZ correlation was markedly larger than half that of the MZ correlation underscoring the presence of a common environmental component. Both the sex-adjusted ACE and AE models accounted well for the data. However, the observed difference in DZ–MZ correlations (that is, the DZ correlation was markedly larger than half that of the MZ correlation) strengthened and emphasized the presence of a common environmental component favoring the ACE model as the most reasonable and descriptive model, providing the most likely inference of the data. The nonadditive component was not significant in any model; however, A could not be ignored, as fixing A to zero produced a significantly worse fit (CE model).

#### Cumulative heritability

**Fig. 4:**
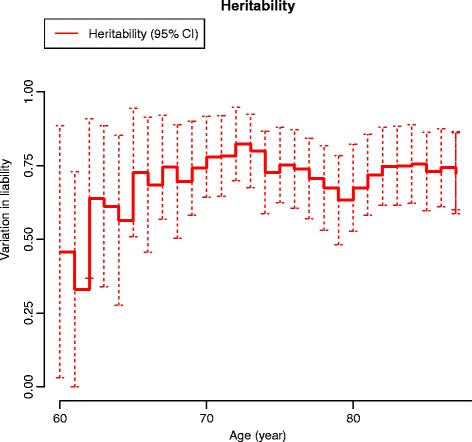
Heritability of THA by age additive genetic factors only. Sex-adjusted variation in liability to THA from additive genetic factors by age. *CI* Confidence interval

**Fig. 5:**
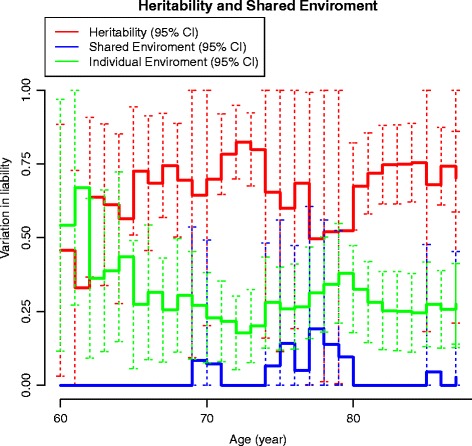
Heritability of THA by age, including the ACE model. Sex-adjusted variation in liability to THA from additive genetic (*red line*), common (*blue line*) and unique (*green line*) environmental factors by age. *CI* Confidence interval

The sex-adjusted heritability on age is presented in Fig. 4. In Fig. 5 the age-related changes in the variance components of A, C and E are displayed. The genetic influence increased from 60 to approximately 75 years of age. These findings were in accord with both the cumulative incidence and the age-associated increase in the proband-wise concordance rates reflecting an association between increasing age and increasing genetic influence. The influence from common environmental factors appeared hardly detectable in younger individuals, but more prominent in the elderly and old.

## Discussion

### Principal findings

Our study is the largest twin study with the longest follow up as to disentangle genetic and environmental factors in symptomatic primary hip osteoarthritis leading to THA. To our knowledge this is the first study on twins from nationwide population-based twin data in a competing risk setting including both same-sex and opposite-sex twin pairs. The cumulative incidence showed an increasing risk from 50 years of age, increasing four- to fivefold at the age of 85 years. After adjustment for sex and age our study showed a significant additive genetic influence attributable to 47 % of the variation in the population liability to THA due to primary hip OA; common and unique environmental factors accounted for 21 and 32 % of this variation, respectively. However, in terms of family factors, that is the combined influence of common genes and shared environment, these factors accounted for 68 % of the population liability to THA. We found no evidence of a sex-specific genetic effect, as the tetrachoric correlations in the SSDZ twin pairs did not exceed that of the OSDZ twin pairs [19].

Further, our graphical presentation of the cumulative incidence, proband-wise concordance rate on age, and heritability on age strongly indicates an association between increasing age and increasing genetic influence, in particular from 60 to 75 years of age.

### Strengths of our study

In our study both same-sex and opposite-sex twin pairs were included providing additional information [19], in contrast to the previously published studies on twins [20–22]. In each model the basic assumption in the CTD that the normally distributed underlying liability to a disease or trait in a population is caused by genetic and environmental factors was met, expressed as equal prevalence in MZ and DZ twin pairs [19, 30]. We included saturated models with broad sense heritability, which includes both additive and nonadditive genetic factors [37–39]. The advantage of modeling broad sense heritability is that it is simple, and loses no information as all co-variances are treated as free parameters, and hence expresses the proportion of the liability to THA attributable to genetic factors. As a case we used a twin who had THA independent of co-twin status, which represents a clinically well-defined outcome [5]. These patients represent a heavy and significant disease burden contrary to cases based on conventional radiographic examination. We used this case definition as studies defining hip OA cases from radiographic findings with or without symptoms may encounter some difficulties in defining their cases as disease severity varies, and the correlation between symptoms or clinical presentation and radiographic findings generally is poor [2–5, 10]. Population-based nationwide registers are highly informative and feasible in follow-up studies involving competing events, and may provide the opportunity to observe competing events of a particular interest for several years in time, and for each sex separately. In our study the events of interest were the occurrence of THA and death, as death becomes a competing risk affecting the possibility of becoming a case. In general, twins are representative of the general population with respect to common complex diseases [40]; correspondingly, our twin population with a THA did not differ from the non-twin population (77 versus 76 %). However, a certain degree of misclassification with respect to the diagnosis might occur, but would be nondifferential as we have no reason to believe that such a misclassification would differ between twins and non-twins. The use of the CIF in our study reflects the age-related probability or risk for THA taking right censoring and the event of death into account. This methodology is increasingly being applied in medical research, and is preferable to the Kaplan-Meier method, which assumes that the events are independent, hence overestimating the event of interest by censoring the other event(s) [31, 32]. The cumulative incidence curves are intuitively appealing and easy to understand. Finally, the censored nature of the data was accounted for by applying a liability threshold model extended with an inverse probability weighting of being censored [36].

### Limitations

Our study has some important limitations. We did not include radiographic findings, but the diagnosis of hip OA in the DHR has previously been satisfactorily validated with respect to completeness and PPV [28, 29]. However, during the last decade an increasing acuity regarding the premise of the diagnosis “primary” or “idiopathic” OA of the hip has unfolded. These deliberations have been stimulated by the theory proposed by Ganz et al. in 2003, claiming that most cases of primary hip OA are in fact secondary due to morphological abnormalities of the hip joint uniformly termed femeroacetabular impingement (FAI) [41]. The hypothesis states that these, sometimes subtle changes are not the consequences of OA, but rather that these changes indeed are risk factors for OA of the hip. Two types of FAI have been defined, the cam-type and the pincer-type. Recently published studies, however, indicate that cam-type FAI may increase the risk of hip OA, but, importantly, most cam-type deformities remain asymptomatic for a lifetime with a PPV of 6–25 % for developing hip OA [42]. Pincer-type, on the contrary, has recently been reported not to constitute a risk for development of hip OA [43]. Further, there are as yet few studies examining the prevalence of radiographic changes in symptom-free individuals and the lack of uniform diagnostic criteria for cam-type as well as pincer-type and of long-term prospective follow-up studies makes it difficult to determine the natural history of FAI and its relation to hip OA [44]. In a long-term follow-up study, Hartofilakidis et al. examined 96 hips in 96 asymptomatic patients in the age range from 16 to 65 years with radiological signs of FAI cam, pincer, and mixed cam–pincer types; 82.3 % of the hips remained free of OA for a mean of 18.5 years (10 to 40), the remaining 17.7 % developed OA at a mean of 12 years (2 to 28), but without any statistically significant difference in the rates of OA development among the FAI groups. The only predictive covariate for hip OA development was the presence of idiopathic OA in the contra-lateral hip, causing the authors to conclude that a large proportion of hips with FAI do not develop hip OA in the long term [45]. The FAI, neither cam- nor pincer- or mixed-type, have as yet been classified in the ICD10, but usually are registered by the diagnosis DM25.5 “Pain in the hip” or DM25.8 “Other diseases of the hip”. This lack of transparency may constitute a risk in our study to have included twin cases diagnosed as primary hip OA, but rightly were secondary OA due to FAI. This, we believe, might have occurred but to a limited extend, as less than 6 % of the patients in the DHR 1995–2010 cohort are younger than 50 years of age and symptomatic FAI is primarily present in younger individuals often engaged in high-impact physical activities. However, some degree of diagnostic misclassification has undoubtedly resulted, but this would be evenly distributed between zygosity groups and hence of the nondifferential type producing slightly weakened heritability estimates by reducing the number of true total cases resulting in wider 95 % CIs. A slightly higher prevalence estimate might also have ensued; however, this would not intimidate the basic assumption in the twin study design as these prevalence estimates would be evenly distributed between the zygosity groups. The CTD is the most used method to disintegrate a particular disease or trait into its genetic and environmental variance components. However, it has long been realized that some assumptions in the CTD can lead to bias in the parameter estimate. This problem has been discussed in great detail elsewhere [37–39], but briefly here, in the normal method of fixing parameters in the CTD, either D or C is fixed to zero in the biometric modeling. This will often cause the additive genetic component to be overestimated at the expense of the common environmental component, which is underestimated. The maximum bias in the common environmental co-variation estimate occurs in the AE model, where C is fixed to zero, when the DZ correlation exactly equals half the MZ correlation, which is the case in our sex-adjusted AE model [37–39]. Hence, the common environmental component C in the ACE model appears as an additive genetic component in the AE model. In view of this conservatism in the CTD to fully differentiate common environmental from additive genetic effects, we find it reasonable, and even right, to blunt Occam’s Razor, and claim the ACE model as the most likely interpretation of our data.

In our study we could not adjust for BMI and occupational exposures, as the registers included did not hold this information. However, studies on BMI as a risk factor for THA has been subject to some ambiguity [6, 10, 13]. Several studies on occupational exposure and hip OA leading to THA have been published [11, 14, 15], and some epidemiological studies have pointed at certain professions, i.e., farming and fishing, but primarily in males [5, 14]. A recent study on occupational cumulative physical exposure, and risk of THA due to OA, reported a cumulative exposure to heavy physical work load as a risk factor, but only in males [15]. In the study by Franklin et al., no relation between occupation and THA was observed in women, but a significant risk was found in male farmers and fishermen [5]. However, a recent study by Andersen et al. reported an increased risk of THA in female healthcare assistants and farmers based on a Danish ational cohort [14]. Occupational exposure to heavy physical work tasks may moderately increase the risk of hip OA leading to THA [11, 14, 15], but, so far, no published twin study on symptomatic or radiographic confirmed hip OA has suggested a reduction in the genetic influence by adjustment for hip joint straining occupational exposure.

### Context

Previously published family and sibling studies have pointed at a major family predisposition to advanced primary hip OA [16–18]. Our study supports this notion by our finding of an additive genetic component of 47 %, and a common environmental component of 21 % accounting in all for 68 % of the variation in the population liability to this disease. None of the few previously published twin studies on hip OA using the CTD seems to relate to the methodological weaknesses with respect to the existence of a common environmental component. As Keller and Coventry point out, the effects of the common environment are often underestimated in CTD studies, and the estimates reported very seldom include the common environmental variation model fit statistics of the examined phenotype [37, 38]. The few published twin studies on hip OA were cross-sectional in design and based on healthy female twins with radiographic hip OA or male twins with self-reported hip replacement due to OA, with heritability estimates, all AE models, from 28 % to 58 %, respectively. Our sex-adjusted heritability estimate of 47 % is in line with these findings, and our finding of a common environmental component of 21 % may represent the effect of taking right censoring into account in our follow-up study [36].

Common environmental factors include shared family life with respect to upbringing and cohabitation potentially inflicting a negative association between family factors and the risk of hip OA in later life. However, a weakness in the CTD is that it cannot discriminate the various sources of the shared environment [19]. Despite this limitation, recent twin studies have reported a significant contribution from the shared environment in the correlation between high education and low BMI (and the reverse), and low educational attainment has been reported to be significantly associated with severity of physical impairment in symptomatic hip OA [46, 47]. It is interesting here to relate to the study by Franklin et al. in which an intriguing finding was that 83 % of farmers were found to be sons of farmers [5].

Hip OA is highly associated with age, but does not manifest in all individuals even of advanced age, and symptoms may vary from none to severe pain [3–5]. Hence, age-associated joint or cartilage changes may serve as a basis for initiation of hip OA triggered by risk factors such as genetic susceptibility, shared influence from the family environment or the individual environment, i.e., occupational exposure to heavy lifting has recently been reported to be associated with early hip structural abnormalities in 3.0 T magnetic resonance imaging [48]. Interestingly, a substantial genetic influence on the progression of joint space narrowing and osteophytes, as mirrored in radiographic OA as cartilage breakdown, has previously been reported [49]. Correspondingly, clinical primary hip OA progression, including radiographic signs and symptoms, has recently been reported to be influenced by genetic predisposition [16]. Interestingly related to these findings, chondrocyte senescence and resulting changes due to aging in the cartilage matrix produce deteriorating functioning and loss of joint cartilage, a key feature in OA [50]. In this perspective, it is interesting that Hjelmborg et al. in a large population-based twin study reported an increasing genetic influence on human lifespan particularly after the age of 60 [51], in particular as both the ageing population and the OA prevalence is highly correlated and increasing. As an intriguing comparison, a large twin-based follow-up study recently reported the age associated changes in bone mineral density to be highly heritable in younger women, but the heritability decreased with increasing age to disappear after the age of 65 years [52]. These findings indicate that the genetic influence on human traits or diseases may be of a more dynamic rather than static nature.

### Implications

The CIF provides a ready instrument to assess the risk of THA, and may be helpful to clinicians as well as to healthcare planners as the proportion of the elderly and old is increasing in western populations. For instance, Kurtz et al. estimated that the numbers of primary THA will grow 174 % in the USA from 2005 to 2030 [53]. Our study points out that particularly important in this development are family factors comprised of genes and shared environment. The graphical presentation of the age-associated heritability estimates indicates that in the elderly individuals genetic factors are important, and increase with ageing; correspondingly, the individual environmental factors decrease from 60 years of age. Prevention is an economically sound instrument and, as the impact of genes and environment is significant, we propose that preventive measures should be launched early in occupational life, and should focus in particular on individuals with a genetic predisposition such as a family history of hip OA. Our finding that the SSDZ correlations did not exceed that of the OSDZ correlation did not support the existence of sex-specific genetic effects in this severe form of hip OA, which may imply that neither sex-specific autosomal nor X-chromosomal gene expression is of a particular importance. Whether or not sex-specific genetic effects are present does have important implications for future research. Indeed, in recent years, research in sex-interaction effects in human diseases has provided increasing evidence of the existence of sex-specific genetic mechanisms [54]. Our study results may be helpful in future research in the specific genetic architecture by pointing at the association between age and genetic influence, as well as development of preventive strategies.

## Conclusion

Family factors of genes and shared environment are highly significant and account for 68 % of the variation in liability to THA due to primary hip OA. We found an age-associated increase in the genetic influence in the elderly which may explain, at least partly, the age-associated increase in primary OA of the hip leading to THA. The cumulative incidence increased in particular after the age of 50 years extending to a four- to fivefold increase at the age of 85 years with no significant sex difference. Studies on the genetic and environmental mechanisms of the age-associated increase as well as the specific genetic architecture of hip OA is warranted, in particular genetic epidemiological studies examining the genetic and environmental influence on FAI and its relationship to hip OA.

## Abbreviation

BMI: Body mass index
CI: Confidence interval
CIF: Cumulative incidence function
CTD: Classical twin design
DHR: Danish Hip Arthroplasty Register
DTR: Danish Twin Register
DZ: Dizygotic twin
FAI: Femeroacetabular impingement
ICD10: International Classification of Diseases 10
MZ: Monozygotic twin
OA: Osteoarthritis
OSDZ: Opposite-sex dizygotic twin
PPV: Positive predictive value
SSDZ: Same-sex dizygotic twin
THA: Total hip arthroplasty
